# Physical-based therapy for the treatment of functional dyspepsia: a systematic review

**DOI:** 10.1007/s12328-026-02288-2

**Published:** 2026-02-11

**Authors:** Peter Liptak, Jakub Hoferica, Marek Vojtko, Peter Banovcin

**Affiliations:** Clinic of Internal Medicine-Gastroenterology, University Hospital in Martin, Jessenius Faculty of Medicine in Martin, Comenius University, Kollarova 2, 03601 Martin, Slovakia

**Keywords:** Functional dyspepsia, Therapy, Review, Exercise, Physical

## Abstract

Functional dyspepsia is a relatively common gastrointestinal disorder characterized by upper abdominal discomfort, bloating, early satiety, and nausea in the absence of any identifiable organic cause. As there is no known causal cure, this disease significantly affects the quality of life of patients. It has been reported that functional dyspepsia could be related to lower exercise levels. As exercise and manual manipulation could result in a lower cost of health care than medications do, it is important to establish whether these types of therapy have a real impact on the alleviation of symptoms. Therefore, we aimed to perform a systematic review with the aim of evaluating the effects of physical treatment options for functional dyspepsia. The study protocol was registered on PROSPERO (CRD42024533101). A total of 12,217 studies were identified across three databases: Embase, PubMed, and CENTRAL. After removing duplicates, abstract screening, assessment for full-text eligibility and further exclusion, five studies were considered for data analysis. The total number of participants in these studies who fulfilled the eligibility criteria and were analysed was 266. Among these patients, 134 received intervention, whereas 132 were in the control group. The analysed studies revealed that this type of intervention could lead to improvements in symptom severity and quality of life in patients with functional dyspepsia compared with controls. These benefits were especially present when exercise was combined with relaxation or biofeedback techniques. However, heterogeneity in interventions and outcome measures limits the strength of conclusions, although no hight risk of bias was identified.

## Introduction

Functional dyspepsia (FD) is a relatively common gastrointestinal disorder from the spectrum of disorders of gut–brain interactions (DGBIs) characterized by upper abdominal discomfort, bloating, early satiety, and nausea in the absence of any identifiable organic cause [1]. The prevalence is approximately 8% of the global population, with significant regional differences [2], and is greater among women than among men [3]. As there is no known causal cure, this disease significantly affects the quality of life of patients [4, 5]. On the other hand, there is a lack of coherent evidence concerning the effect of lifestyle alterations on symptoms of dyspepsia [6]. According to the current consensus of the European Society of Neurogastroenterology and Motility (ESNM), treatment consists of proton pump inhibitors and/or neuromodulate therapy [7]. The American College of Gastroenterology (ACG) and the Canadian Association of Gastroenterology (CAG) joint statements also suggest prokinetics as a therapeutic modality [8]. If Helicobacter infection is present, it must be eradicated [9]. In Asia, fundic relaxants such as buspirone and acotiamide are evaluated in the guidelines but again have a weak grade of recommendation [10]. In Japan, herbal medicine is considered a first-line treatment option [11]. Some patients may benefit from dietary adjustments, e.g., eating smaller portions of food and restricting fat intake [12]. Among nonpharmaceutical treatment options, various psychological interventions and stress management options are suggested, but most of these options have a low quality of evidence and a low level of consensus [13]. The emerging new field of virtual reality as a potential therapeutic option could be a target for further research [14, 15].

Interestingly, physical-based therapies, such as exercise, joga, and massage, are not reflected in guidelines around the world [7, 8, 10, 13]. However, from a pathophysiological point of view, the notion of symptom alleviation by physical therapy in functional dyspepsia could be possible. The positive role of exercise on gut health is well established [16, 17], including its influence on the microbiome [18, 19]. It has been reported that functional dyspepsia could be related to lower exercise levels [20, 21]. Furthermore, there is scarce evidence that massage [22–24] and yoga [25–27] lead to improved gut function and a reduction in symptoms of functional dyspepsia. As exercise and manual manipulation could result in a lower cost of health care than medications do, it is important to establish whether these types of therapy have a real impact on the alleviation of symptoms.

Therefore, we aimed to perform a systematic review with the aim of evaluating the effects of physical treatment options for functional dyspepsia.

## Methods

We report our systematic review based on the recommendation of the PRISMA 2020 guidelines [28], while we followed the Cochrane Handbook [29]. The study protocol was registered on PROSPERO (CRD42024533101), and we fully adhered to it.

### Eligibility criteria

Only randomized controlled trials (RCTs), cohort studies, and case‒control studies were included in this analysis, while case series and case reports were excluded. The eligibility criteria were established via the PICO framework (patients, intervention, comparison, outcome). The patient population comprised individuals diagnosed with functional dyspepsia, presenting either with the epigastric pain syndrome (EPS) or postprandial distress syndrome (PDS) phenotypes, or a combination of both. The diagnosis was required to adhere to the ROME II [30], ROME III [31], or ROME IV [32] diagnostic criteria. The interventions evaluated encompassed various forms of physical therapy, including deep breathing exercises, aerobic and anaerobic exercise, resistance training, yoga, massage, physiotherapy, rehabilitation, biofeedback, and balneotherapy-based treatments. These interventions were compared against control groups with either no physical activity or placebo.

The primary outcome was significant improvement in functional dyspepsia symptoms assessed by patients or healthcare professionals/researchers. Owing to the nature of the data, we assessed the primary outcomes in different forms, such as the Glasgow dyspepsia severity score (GDSS) [33], Depression Anxiety Stress Severity Scale (DASS-42) [34], and visual analogue scale (VAS) [35]. Various types of questionnaires, such as the Short-Form Nepean Dyspepsia Index [36], the Sense of Humor Questionnaire (SHQ-6) [37], the Neuroticism Subscale of the ‘‘Eysenck Personality Questionnaire’’ (EPQ-N) [38], the Telic Dominance Scale [39] or the daily symptom questionnaire, were used along the assessment of drinking capacity, intragastric volume, and gastric emptying. Additionally, the frequency of gastrointestinal symptoms, number of anal gas evacuations, and colonic content measured via abdominal magnetic resonance imaging were measured. One study in perimenopausal women [40], among others, assessed cortisol and estradiol levels and the Pittsburgh Sleep Quality Index (PSQI) [41]. The secondary outcomes included improvements in quality of life (QoL) and the occurrence of adverse events associated with the interventions.

### Information sources

The search was performed on 25.4.2024 in three main databases: Embase, PubMed, and the Cochrane Central Register of Controlled Trials (CENTRAL). No language or other restrictions were applied. Additionally, the references of the included studies were systematically searched via a citation chaser [42]**.**

### Search strategy

To address the lack of a consensus definition and to reduce the risk of missing relevant trials, we deliberately used a broad search strategy. During the systematic search the following search key was used ((functional OR chronic OR uninvestigated) AND dyspepsia OR (rome AND criteria)) AND ((functional OR chronic OR uninvestigated) AND (dyspepsia OR (rome AND criteria)) AND (physiotherap* OR ‘physical’ OR ‘manual’) AND (therap* OR treatmen* OR intervent*) OR breathing OR yoga OR joga OR train* OR hydrotherap* OR balneo* OR ‘spa’ OR manipulat* OR massag* OR exercis* OR (wor* AND out) OR rehabilitat*).

### Selection process

The selection was performed by two independent review authors at Rayyan [43] (P.L. and M.V.) after the duplicates were removed, first by title and abstract and then by full text (considering the eligibility criteria). Cohen’s kappa coefficient was calculated at both levels of selection to measure the interviewer reliability. We assessed eligibility independently according to the predefined criteria, with any disagreements between investigators resolved by a third independent party.

We prespecified that randomized controlled trials, cohort studies, and case–control studies were eligible, however, during title and abstract assessment no observational study (cohort or case–control) met all inclusion criteria and therefore only randomized trials were included in the review. No language restrictions were applied. Of the 433 non-English records screened at title and abstract, 0 were assessed in full text. Reasons for exclusion mirrored those of English-language reports, and no non-English study met all eligibility criteria.

### Data collection process

From the eligible articles, all the data were collected independently by two authors (P.L. and M.V.). Disagreements were resolved by J.H. All the data were manually collected and entered into an Excel spreadsheet (Office 365, Microsoft, Redmond, WA, USA) in preparation for analysis.

### Data items

The following data were extracted: first author, year of publication, study design and period, geographic location, number of centers, age, and sex.

In addition, we extracted data on the total number of patients and type of intervention, including duration of therapy and number of sessions, from each arm (intervention/control). Rome diagnostic criteria and definitions of the outcomes of interest were also extracted. (type of questionnaire, type of symptom relief assessment).

### Study risk of bias assessment

The ROB-2 tool [44] was used for ROB assessment by two independent investigators (MV and PL). Independent third parties (JHs) resolved all disagreements. The results were visualized via the Robvis tool [45].

## Results

### Search and selection

A total of 12,217 studies were identified across three databases: 6,827 from Embase, 4,291 from PubMed, and 1,099 from CENTRAL. After removing duplicates, 9,071 unique records remained for title and abstract screening. Among these, 25 studies were assessed for full-text eligibility. Of these, 14 were excluded for prespecified reasons (ineligible intervention, outcome, overlapping population, study design restrictions) and 5 were ongoing or not yet fully published studies. Additionally, one study focused on a specific profession and gender, limiting its generalizability leaving 5 randomized controlled trials for qualitative synthesis. Cohen’s kappa for title/abstract and full-text screening was 0.89 and 0.91, respectively.

**Fig. 1:**
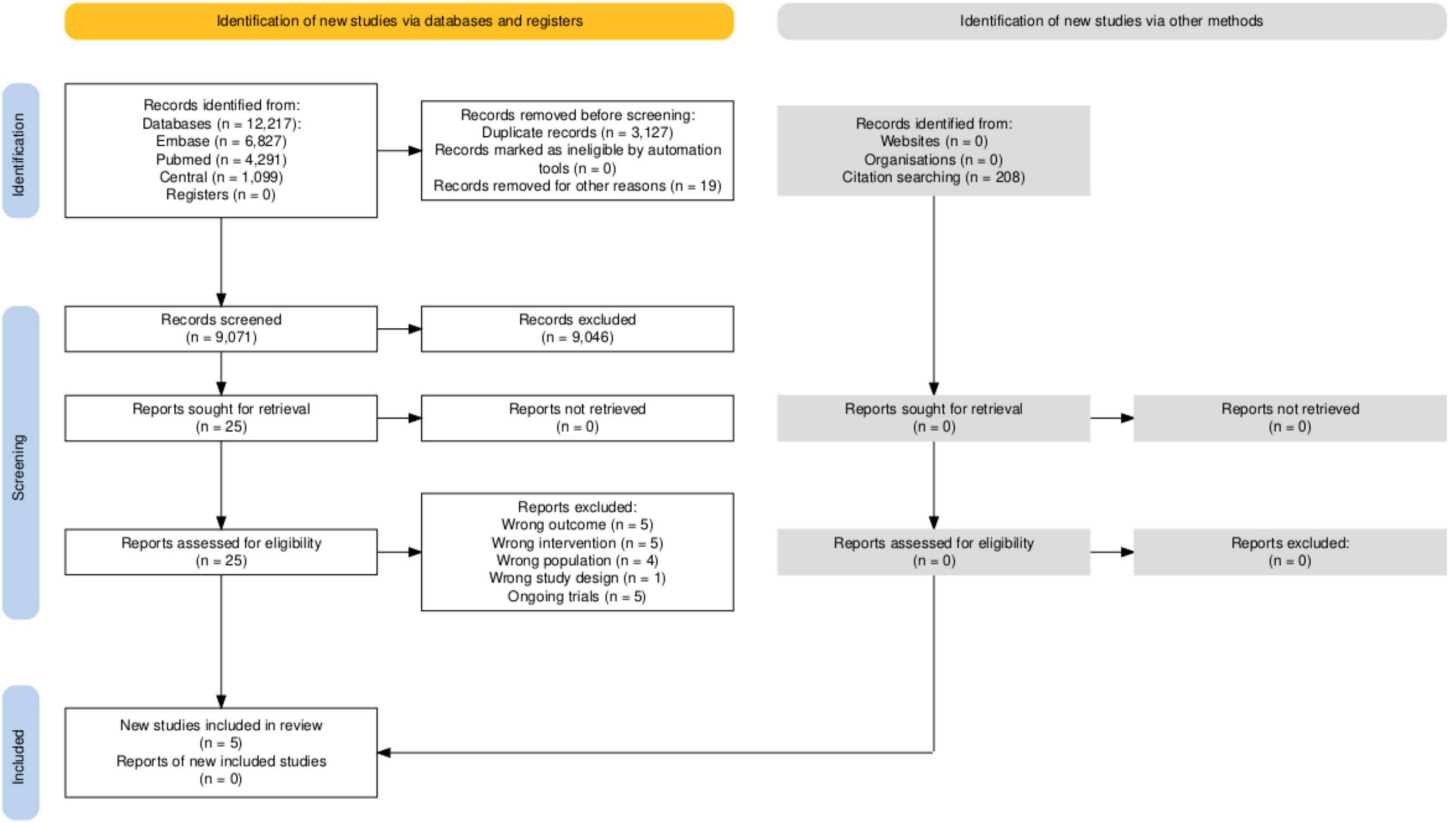
PRISMA 2020 flowchart representing the study selection process

In addition, we identified 208 records through citation chasing, no study was sought for retrieval and was eligible for data extraction. For more details on our search and selection process, see the PRISMA flowchart (Fig. 1).

### Basic characteristics of the included studies

The 5 eligible studies examining physical-based therapy for the treatment of functional dyspepsia were published from 2007 to 2022 and included sample sizes ranging from 50 to 112 participants. The total number of participants in these studies was 427, of whom 266 fulfilled the eligibility criteria and were analysed; 134 received physical-based therapy, whereas 132 underwent a control intervention. The participants’ ages ranged from 18 to 63 years, with a predominance of female patients (215 patients from 266 total). Patients presented with EPS (epigastric pain syndrome) or PDS (postprandial distress syndrome) or their overlap. All the studies were conducted in different countries and were predominantly single-center studies, with the exception of one parallel trial at 2 referral centers in Spain [46]. Only single- or double-blinded randomized controlled trials were included, with 2 adhering to the Rome IV criteria, 2 adhering to the Rome III criteria, and 1 following the Rome II criteria.

The baseline characteristics of the patients included in the analyses are detailed in Table 1. In Table 2, we summarize the ongoing studies.

**Table 1:** Basic characteristics of included studies

References	Country (number of centres)	Study Design	Number of analysed patients (female% of total)	Intervention group	Control group	Rome criteria	Outcome
Rane et al. [47]	India Single centre	Single-blind prospective randomized controlled trial	72 (44,4%)	Exercise 5 times a week, 6 weeks, 30 min session	Standard care (Pantoprazol 40 mg once daily)	IV	GDSS DASS-42 VAS
Hjelland et al. [49]	Norway Single centre	Single-blind prospective randomized placebo controlled trial	40 (80%)	6 breaths/min 5 min daily with vagal biofeedback device. 4 weeks	Not specifically mentioned, **supposed** standard undisclosed care	II	SF-NDI SHQ-6 Telic Dominance Scale EPQ-N RSA SC Gastric empting Intragastric volume Drinking capacity
Koklu et al. [48]	Turkey Single centre	Single-blind prospective randomized placebo controlled trial	44 (95%)	Vacuum interferential current of frequency 4 kHz,4 weeks, 12 × 15 min sessions	Placebo	III	ITT analysis( questionnaire) PP( questionnaire)
Huaman et al. [46]	Spain Two centres	Double blind randomized parallel controlled trial	50 (86%)	Correction of dyssynergic defecation by 2–3 sessions of biofeedback combined with instructions for daily exercising	Fiber supplementation- 3.5 g plantago ovata per day	IV	Daily symptom questionnaire Anal evacuations Colonic content (MR)
Ismail et al. [40]	Egypt Single centre	Double blind randomized controlled trial	60 (100%)	Aerobic exercise(5 sessions per week) plus BRT for 40-min (diaphragmatic breathing and progressive muscle relaxation applied for 20 min in the morning and evening) + stardard care	BRT only for 40-min (diaphragmatic breathing and progressive muscle relaxation applied for 20 min in the morning and evening) + stardard care	III	GDSS Cortisol- primary result VAS Estradiol PSQI DASS-42

*GDSS* Glasgow dyspepsia severity score, *DASS-42* Depression Anxiety Stress Severity, *VAS* Visual analog scale, *SF-NDI* Short-form Nepean Dyspepsia Index, *SHQ-6* Sense of Humour Questionnaire, *EPQ-N* Neuroticism Subscale of the ‘‘Eysenck Personality Questionnaire’’, *RSA* Respiratory Sinus Arythmia, *SC* Skin conductance, *ITT* Intention-to-treat analysis, *PP* Per-protocol analysis, *PSQI* Pittsburgh sleep quality index, *BRT* Benson relaxation therapy

**Table 2:** Basic characteristics of ongoing studies

First Author	Study start	Study	Country	Study design	Number of patients	Intervention group	Control group	Outcome
Wang	2020	Unknown status	China	Interventional study	260	Exercise: Jogging or cycling ≥ 5 days/week, 30–60 min/d	Behavioral: Maintaining original lifestyle	Weekly global symptoms assessment12 weeks
Arisandy	2024	Recruiting	Indonesia	Interventional parallel study	54	Integrated visceral and spinal manipulation (In-ViSMa)	Integrated Abdominal Massage and Spinal Manipulation (In-VaSMa)	LDQ PSS API PSQI SF-NDI
Rossi	2009	Unknown status	Italy	Interventional study	50	4 Visceral Manipulation visits + PPIs and/or Domperidone for 4 week	PPIs and/or Domperidone for 4 weeks,	VAS
Bhavanani	2019	Not yet recruiting	India	Randomized, parallel group trial	152	Yoga therapy along with standard medical management. Guided session once a week and everyday practice of yoga	Standard medical management	GSRS and Dyspepsia Questionnaire H.Pylori stool antigen test PSS, GAD-7 and PHQ-9
NA	2018	Completed	China	Interventional parallel study	94	Transcutaneous auricular vagus nerve stimulation: taVNS	Placebo: tnVNS	FD Symptoms Index, FDDQL, HAMA, HAMD, SDS

*LDQ* Leeds Dyspepsia Questionnaire, *PSS* Perceived Stress Scal, *API* Abdominal Pain Index, *PSQI* Pittsburg Sleep Quality Index, *SF-NDI* Short Form-Nepean Dyspepsia Index FD Symptoms Index, *FDDQL* Functional Dyspepsia Quality of Life Scale, *HAMA* Hamilton Anxiety Scale, *HAMD* Hamilton Depression Scale, *SDS* Self-Depression Rating Scale, *GSRS* Gastrointestinal Symptom Rating Scale, *PSS* Perceived Stress Scale, *GAD-7* The Generalised Anxiety Disorder Assessment, *PHQ-9* Patient Health Questionnaire-9

#### Intervention groups and controls

There was significant heterogeneity in the types of physically based interventions in the evaluated studies, but we analysed three trials (Rane [47], Ismail [40], and Huaman [46]) that used aerobic exercise alone or in combination with other physically based therapies compared with the control groups. In the study by Rane et al. [47], the intervention group performed moderate aerobic exercise for 30 min per session, 5 times a week for 6 weeks, along with standard care. The aerobic exercise combined 20 min of brisk walking, 5 min of warm-up and cool-down, stretching, and deep breathing exercises. Ismail et al. [40] chose a combination of walking on a treadmill session, Benson relaxation therapy, and a daily dose of proton pump inhibitors as suitable interventions. Each session lasted 30 or 40 min (in the second month), with a 5-min warm-up and cool-down period, 5 times per week. Both the intervention and control groups were subjected to 20-min morning and evening Benson’s relaxation therapy (BRT), which consisted of diaphragmatic breathing and progressive muscle relaxation daily for 8 weeks. In a parallel trial from Spain [46], the effect of the correction of dyssynergic defecation in patients with overlapping functional constipation on symptoms of functional dyspepsia was evaluated. Patients were assigned to two intervention groups: first, they underwent defecation correction via biofeedback in combination with nonspecified daily exercise; second, they received fibre supplementation. Biofeedback training (2–3 sessions) was conducted in the office via manometric monitoring to guide anal relaxation during straining. Sessions lasted 30–45 min over 6 weeks, with 2 weeks of pretreatment and 4 weeks of treatment.

A trial performed by Koklu and colleagues [48] administered interferential current using four electrodes, with a carrier frequency of 4 kHz and a beat frequency sweep covering 80–150 Hz. The study lasted for 4 weeks, and both the IFC and placebo groups received 12 × 15 min of treatment with either active or sham IFC.

Finally, a study from Norway by Hjelland et al. [49] investigated the effect of breathing exercises combined with vagal biofeedback. For 4 weeks, the participants practiced breathing exercises of 6 breaths per min for 5 min daily with vagal biofeedback. Patients in the control group were informed about FDs, and standard undisclosed care was provided.

#### Outcomes

Owing to heterogeneity in the interventions used in the analysed trials, improvements in symptoms of functional dyspepsia were also reported by various means.

Most of the studies [40, 46–48] have assessed the benefit of physical-based therapy via questionnaires. Various questionnaires were used, but most patients (132) were assessed by the Glasgow Dyspepsia Severity Scale (GDSS), Depression Anxiety and Stress Scale (DASS-42) questionnaires, and visual analogue scale (VAS) [40, 47], with a significant difference in favour of the intervention group. Studies that used other questionnaires, such as daily symptom questionnaires [46], the SF-NDI [49] or nonvalidated questionnaires [48], also reported significant differences between the intervention and control groups in favour of the intervention group.

The study by Ismail reported significant improvements across all measured outcomes in both the intervention and control groups, except for serum estradiol levels in the control group. These findings suggest that BRT, with or without exercise, could have a beneficial effect on functional dyspepsia symptoms. The remaining studies [46, 49] revealed increased drinking capacity after treatment with breathing exercises combined with vagal biofeedback and that the correction of dyssynergic defecation was associated with a significant reduction in postprandial fullness, distension, and discomfort/pain, further supporting the possible positive role of physical-based therapy in the management of functional dyspepsia.

#### Risk of bias assessment

**Fig. 2:**
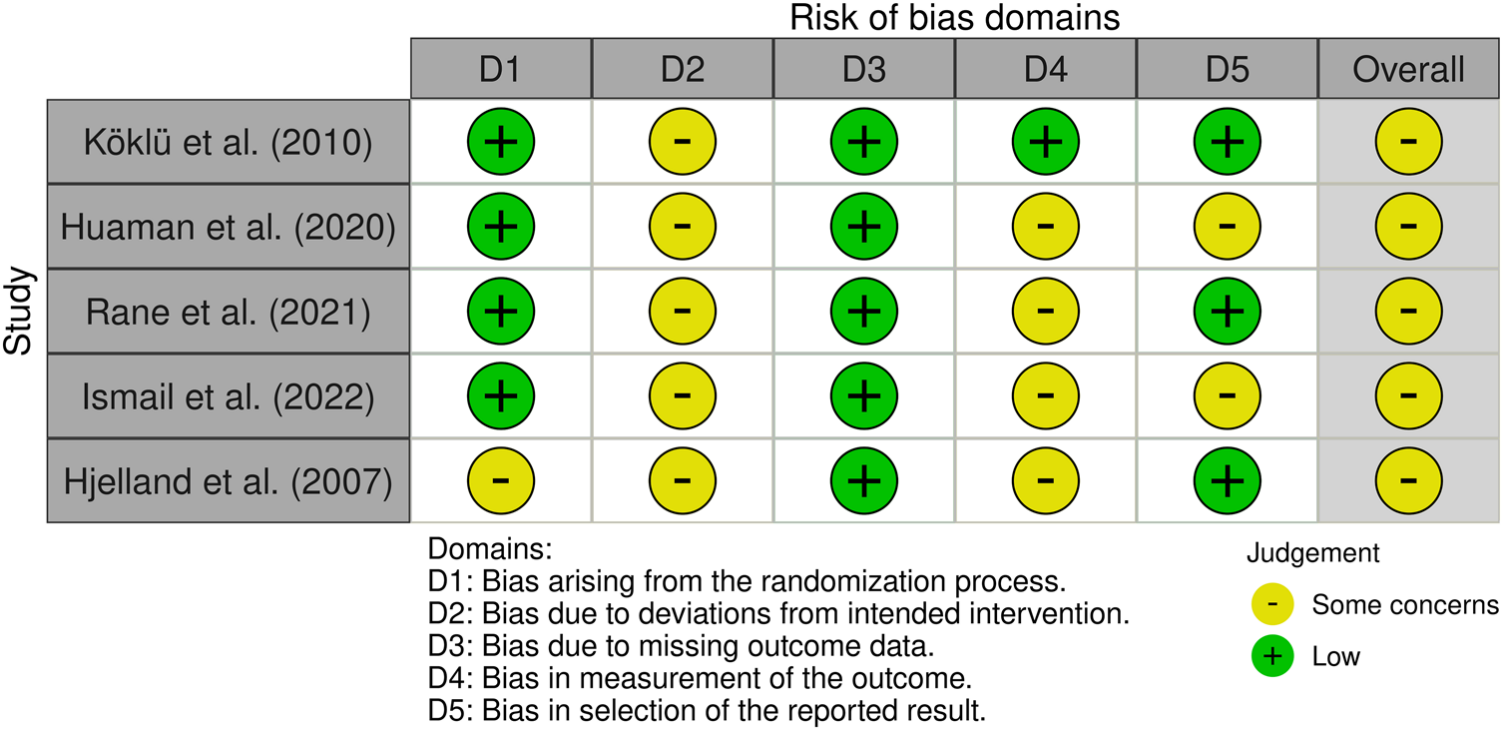
Risk of bias domains. Overall, all of the presented studies present some concern of bias. However, no high bias risk was identified

**Fig. 3:**
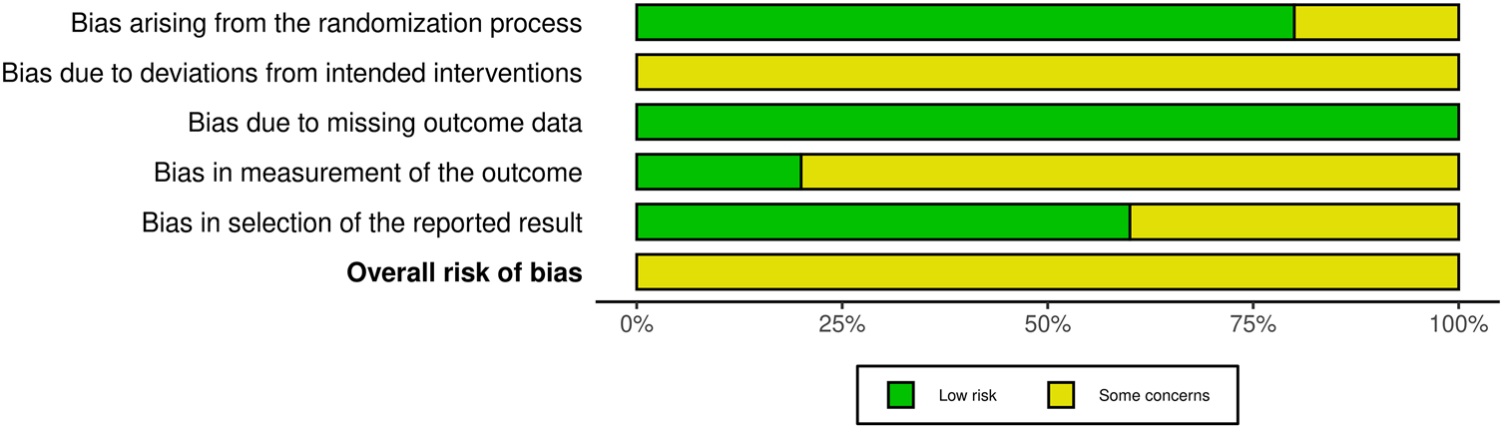
The risk of bias in various areas is presented as a percentage. Overall, all of the presented studies present some concern of bias. However, no high bias risk was identified

All publications manifested some concern regarding the level of risk of bias based on ROB2 tool. No low or high ROB was detected. The elevated ROB raised mainly from domains of deviations from intended interventions and measurement of the outcome. For more details, see Fig. 2 and Fig. 3.

## Discussion

Functional dyspepsia is a common gastrointestinal disorder characterized by upper abdominal discomfort, bloating, early satiety, and nausea in the absence of any identifiable organic cause. It significantly impacts quality of life and is often exacerbated by stress, hormonal changes, and lifestyle factors. While treatment traditionally focuses on pharmacological interventions, increasing evidence supports the potential benefits of exercise as a nonpharmacological approach to managing FD. It has been shown that exercise intervention positively promotes gut motility [50, 51]. Although not correlated with symptom occurrence and impaired accommodation [52], gastric dysmotility is considered one of the key factors in the pathophysiology of functional dyspepsia. Among the other putative pathophysiological factors are currently widely accepted and recognized visceral hypersensitivity [53], alterations in the duodenal microbiota [54], and discrete subclinical duodenal inflammation [55] associated with the possibility of a so-called leaky gut [56, 57]. Emerging evidence also highlights the significant role of immunoactive cells, namely, eosinophils [58, 59] and mastocytes [60–62], as primal promoters of FD etiology. The evaluation of the presence and subsequent eradication of *Helicobacter pylori*, if present, represents a cornerstone of therapeutic approaches [63]. Furthermore, proton pump inhibitors, neuromodulators and prokinetics represent relatively effective medications according to the specific phenotype of functional dyspepsia [64–66]. Other therapeutic approaches, including diet modification, psychological interventions, hypnotherapy, herbal medicine, and traditional medicine, do not have sufficient and widely accepted evidence of efficacy [7, 13]. The important factor is to individualize the therapy [67]. Importantly, however, a substantial portion of patients do not report adequate symptom relief after standard, pharmacological intervention [68].

The prevalence of functional dyspepsia was inversely associated with exercise intensity in a relatively large Chinese cohort study [69]. Aerobic exercise is defined as any activity that uses large muscle groups that can be maintained continuously and has a natural rhythm [70].

Moderate exercise is defined as an activity that is performed in the range of 40–60% of maximum oxygen uptake (VO2 max) [71]. Exercise is associated with stimulation of gut motility, improving the whole gut transit time [72], which could have positive implications for symptoms of functional dyspepsia, as one of the possible contributing pathophysiological factors of FD could be impaired gastric emptying [73, 74].

In five randomized trials considered for the final data evaluation in this systematic review, several different physical-based approaches were associated with improvements in dyspepsia symptoms and/or disease-specific quality of life when compared to control group. These interventions could be grouped by their physiological mechanism of action.

### Exercise-based therapies

The exercise-focused trials suggest that well defined and structured physical activity may improve dyspepsia symptoms and disease-specific quality of life through complementary pathways. In the study by Rane et al. [47], moderate aerobic training added to standard care was associated with better combined symptom scores and mental state measures. This could be a biologically reasonable pattern functioning via modest acceleration of gastric emptying and/or whole-gut transit, improving gastric accommodation, and attenuation of stress-related intensification of discomfort. A second trial, Ismal et al. [40], implement aerobic exercise within a program where both groups received structured Benson relaxation technique. Clinically relevant improvements across outcomes in both arms, specifically in case of the addition of regular physical activity, point toward autonomic and perceptual mechanisms that could be targeted in the disease management in order to maximalize the chance of positive therapy outcome.

### Interventions targeting autonomic interactions

The relationships between stress and various disorders of gut–brain interactions have been thoroughly described before [75–78]. Furthermore, various aspects of abnormal psychological responses, such as depression and anxiety, have been associated with DGBIs and functional dyspepsia in particular [79]. This resulted in the application of the principles of relaxation and breathing techniques to the treatment of DGBIs in the experimental setting [80].

Interventions aimed at vagal modulation and arousal regulation also showed positive trends. In a study performed by Hjelland et al. [49], slow diaphragmatic breathing practiced with simple vagal biofeedback was linked to greater drinking capacity and better quality of life compared with control group. Although heart-rate-variability indicators did not change steadily, the clinical improvements are consistent with a probable pathophysiological pathway involving enhanced gastric accommodation and reduced visceral hypersensitivity in these patients. For practical implications, it is important to note that the authors did not sufficiently describe the patient group, so there is no information about previous or concomitant PPI or H2 blocker treatment. Therefore, it is not possible to conclude whether the positive effect of biofeedback could be obtained as an add-on to standard therapy or if it represents a standalone therapeutic modality. In a population with overlapping constipation and dyssynergic defecation, Huaman el al. [46] demonstrated that pelvic-floor biofeedback reduced postprandial fullness, distension, and pain relative to fibre supplementation. This supports the hypothesis that correcting motoric dysfunction within the distal parts of gastrointestinal tract can secondarily alleviate dyspeptic symptoms in selected patients. This present an important clinical implication as these findings emphasis the prevalence of overlap of symptoms of various disorders of the gut‒brain interaction that seems to be an emerging topic in the field of neurogastroenterology and motility [81]. The relationship between constipation and functional dyspepsia is yet another example of a so-called DGBI symptom cluster. This fundamental shift in the view of these disorders is acknowledged and implemented as a clinical consensus [82].

### Peripheral nerve stimulation

Evidence for non-invasive neuromodulation remains scarce but notable. The trial by Köklü et al. [48] using interferential currents reported improvements in early satiety, bloating, and heartburn in the intervention group compared to the group with sham therapy. This is a consistent finding with knowledge of peripheral modulation of enteric/autonomic signalling and nociceptive pathways. Interpretation, however, is reserved by non-standardized symptom assessments and incomplete reporting of stimulation parameters, underscoring the need for harmonized protocols.

An important limitation of this review is the absence of a universally accepted definition of “physical-based therapy”. Our working definition, although specified a priori, remains somewhat arbitrary and may not fully agree with how every scientist or clinician classify specific modalities. This ambiguous concept may have influenced both factors of review process, that be interventions that were included and the final number of eligible studies. All this could be present despite our intentionally broad search strategy.

For this systematic review we did not consider the East Asian characteristic methods of therapy for example acupuncture. This is solely due to its limited availability in general in geographical regions outside East Asia with questionable level of expertise of practitioners in so called western countries (cases of mistreatment by unlicensed acupuncture providers in these are known in public). To provide most universal data with possibility of broad clinical application we decide to consider therapies that are used or could be used widely with certain degree of expertise.

The results of the studies analysed in this systematic review highlight the potential beneficial effects of physical therapy in patients with functional dyspepsia. However, owing to the high degree of clinical heterogeneity among these studies in terms of both interventions and outcome measurements, it is not possible to adequately evaluate the true effect size of physical therapy on functional dyspepsia although no high risk of bias was identified. This heterogeneity prevented us from mathematically synthesizing the available evidence; therefore, no clear conclusions with a sufficient level of certainty can be drawn from the current literature. Additionally, all included studies raised concerns regarding the risk of bias. Notably, several ongoing studies (see Table 2) may provide further insights into the efficacy of physical therapy for alleviating symptoms of functional dyspepsia upon completion.

Given that current international guidelines for the disorders of gut-brain interaction consider pharmacological therapy as the cornerstone of functional dyspepsia management, physical-based interventions may be considered as an adjunctive method within a guideline-directed treatment strategy. Low-intensity aerobic exercise and structured breathing or relaxation techniques seem particularly suitable for incorporation into standard care, especially in patients with refractory symptoms to the standard therapy. Practical barriers of implementation might include limited availability of appropriately trained personnel, heterogeneity in reimbursement among different health care providers, and limited cost-effectiveness data; however, these may well be alleviated by use of simple and/or standardized protocols and accessible home-based or telemetry supported methods of training. This personalized, multimodal therapy could support stronger patient engagement in disease management and therefore leads to better therapeutical outcomes lowering the overall cost to the health care system.

## Conclusion

Physical therapy could present a potential adjuvant therapeutic modality for functional dyspepsia management, especially for patients unresponsive to standard treatment protocols. However, heterogeneity in interventions and outcome measures limits the strength of conclusions in this systematic review, although no high risk of bias was identified. Further randomized control trials with a high number of participants are needed to confirm these results.
